# Exploring the sources of cervical cancer screening self‐efficacy among rural females: A qualitative study

**DOI:** 10.1111/hex.13840

**Published:** 2023-08-07

**Authors:** Mengyue Zhang, Janet W. H. Sit, Tingxuan Wang, Carmen W. H. Chan

**Affiliations:** ^1^ The Nethersole School of Nursing, Faculty of Medicine The Chinese University of Hong Kong Hong Kong China; ^2^ School of Nursing, LKS Faculty of Medicine The University of Hong Kong Hong Kong China

**Keywords:** cancer screening, framework method, self‐efficacy, uterine cervical neoplasm

## Abstract

**Aim:**

Evidence showed self‐efficacy was relevant to rural females' cervical cancer screening behaviour. However, little is known about sources of self‐efficacy in cervical cancer screening among rural females. This study aimed to explore sources of self‐efficacy in cervical cancer screening among rural females.

**Design:**

A qualitative descriptive study was conducted. Both users and providers of cervical cancer screening services in rural areas of China were recruited through maximum variation sampling.

**Methods:**

Individual semi‐structured interviews through telephone calls were conducted. Data were analysed via six main stages of the framework method, with the social cognitive theory as a reference.

**Results:**

Four main sources were identified, including personal screening experience, hearing about other women's screening experiences, professional health education and consultation, and emotional status. Personal screening experience included enactive mastery of completing the screening behaviour and cognitive mastery of internalisation of the screening. Only the experience of completing cervical cancer screening behaviour was not strong enough to improve self‐efficacy. Cognitive mastery showed more critical influence.

**Conclusion:**

These four sources of rural females' cervical cancer screening self‐efficacy matched with the major sources of self‐efficacy of the social cognitive theory. Cognition was critical to influencing the screening self‐efficacy. Intervention strategies aimed at enhancing rural females' cervical cancer screening self‐efficacy can be developed from these four major sources.

**Public Contribution:**

A registered nurse with rich experience in cervical cancer‐related research and qualitative study was the interviewer of this study. Rural females and cervical cancer screening services providers (healthcare professionals and village staff) were recruited as interviewees. The interview guides were developed by the research team and evaluated by an expert panel including two nurse leaders of gynaecological cancer, one doctor specialised in cervical cancer, and one medical director in a local rural hospital.

## 1 INTRODUCTION

Cervical cancer is a significant global public health issue. In 2020, the World Health Organisation (WHO) reported that cervical cancer is the fourth most frequently diagnosed cancer in females. However, health inequalities contribute to significant disparities in cervical cancer diagnosis rates between rural and urban areas.^1^ Socioeconomic factors and limited available healthcare resources reduce rural populations' access to diagnostic and therapeutic services.^2^, ^3^, ^4^, ^5^ Studies have demonstrated that cervical cancer incidence and mortality were higher among rural‐dwelling females than their urban counterparts.^6^, ^7^, ^8^, ^9^ The American Cancer Society proposed that limited access to early detection and preventive care in rural areas may contribute to these disparities in cancer mortality.^10^ Screening is one of the most effective methods for early detection of cervical cancer.^11^, ^12^ Therefore, cervical cancer screening should be specifically promoted in rural areas with low screening uptake rates.

Cervical cancer screening uptake exhibits marked urban–rural differences across both developed and developing countries. In 2011, cervical cancer screening uptake rates in the United States were 74.6% and 83.9% among rural and urban females, respectively.^13^ In China, with the implementation of a national cervical cancer screening programme in 2009, rural females aged 35–64 years could access free cervical cancer screening services.^14^ This programme significantly improved cervical cancer screening uptake in rural areas. However, compared with the United States, screening uptake in rural China remained insufficient. In 2010, the screening uptake rates among urban and rural females in China were 29.1% and 16.9%, respectively.^15^ In 2013, the screening uptake rate among rural females increased to 22.3%, but remained significantly lower than that among urban females (31.8%).^16^

Studies have shown that self‐efficacy is a critical influencing factor that contributes to increased screening uptake among rural females. A study exploring the relationship between self‐efficacy and Pap smear screening behaviour reported that greater levels of self‐efficacy were associated with increased screening uptake among females.^17^ Similarly, a different study reported that self‐efficacy was a predictive factor for preventive behaviours relating to cervical cancer.^18^ Additionally, studies specifically focusing on rural females have reported that self‐efficacy is associated with cervical cancer screening behaviours. One study conducted in the United States reported that self‐efficacy was an important factor influencing rural breast and cervical cancer screening uptake.^19^ A Chinese study reported that self‐efficacy increased rural females' intention to undergo cervical cancer screening.^20^ Therefore, identifying sources of cervical cancer screening self‐efficacy is an important step in positively influencing rural females' screening behaviours.

Quantitative studies have examined the effects of cervical cancer screening‐related knowledge and awareness on rural females' self‐efficacy levels.^19^, ^20^ However, there is a lack of qualitative studies focused on identifying sources of such screening self‐efficacy. To better understand and design efficient interventions to improve rural females' self‐efficacy, it is necessary to explore and identify relevant information using qualitative methods. Therefore, this qualitative descriptive study aimed to identify sources of self‐efficacy relating to cervical cancer screening among rural females. This study focused on addressing the following questions: (i) Identify the main sources of information that influence rural females' cervical cancer screening self‐efficacy. (ii) Determine how these sources of information work to affect rural females' cervical cancer screening self‐efficacy. (iii) Identify the most and least influential determinants of self‐efficacy. The findings of this study can contribute to formulating strategies to enhance screening‐related self‐efficacy in the future.

## 2 FRAMEWORK METHOD

The framework method, which has been widely used in the management and analysis of qualitative data in health research,^21^ was adopted for data analysis in this study. We adopted the framework method that included both inductive and deductive approaches for data analysis and integration. To apply the inductive approach, we used an open coding method to generate themes and explain patterns in the qualitative data. To apply the deductive approach, the social cognitive theory was adopted as a guideline to provide predetermined codes for data analysis.

Self‐efficacy is a core component of the social cognitive theory, which was developed by Bandura.^22^ According to the theory, self‐efficacy is defined as a person's belief in their own ability to organise and execute specific actions required to attain a desired outcome.^23^ Self‐efficacy is vital for instigating behavioural changes as it influences behaviour patterns.^24^ Social cognitive theory proposes four main determinants of self‐efficacy: performance accomplishment, vicarious experience, verbal persuasion, and emotional arousal.^22^ Social cognitive theory provides a clear framework for the main sources of self‐efficacy, which has been used as a theoretical framework by previous studies focusing on exploring the sources of individuals' self‐efficacy.^25^, ^26^ Therefore, the theory was used as a reference in this study's exploration of sources of rural Chinese women's cervical cancer screening‐related self‐efficacy.

## 3 METHODS

### 3.1 Study design

A qualitative descriptive design was selected, including in‐depth, semi‐structured, individual interviews.

### 3.2 Study settings

Participants involved in this study came from administrative villages largely located in the agricultural areas of Zaozhuang, Shandong province, mainland China.

### 3.3 Participant recruitment

Both users and providers of cervical cancer screening services were recruited for the interviews. Rural females who are eligible for screening can provide self‐related information, and service providers, such as healthcare professionals and village staff, can provide additional perspectives. In the Chinese context, in addition to the local healthcare professionals, the trained staff of the villages' Women's Federation is also involved in providing screening‐related services. These women are trained as lay health workers and promote the national cervical cancer screening programme.

The inclusion criteria of rural females were: (i) females aged 25–64 years; (ii) settled in rural areas with a permanent registered rural address; (iii) having sexual experience; (iv) without an existing or previous diagnosis of cervical cancer or cervical precancerous lesions and without a prior hysterectomy. The exclusion criteria were: (i) being pregnant; (ii) having a medical diagnosis of any serious physical illness or mental or cognitive disorders; (iii) having difficulties with oral expression or communication; (iv) participating in another cervical cancer screening‐related research at the same time. Certified local healthcare professionals responsible for previous or ongoing cervical cancer screening services and village staff involved in the promotion of the national screening programme were also recruited for interviews.

### 3.4 Sampling

Maximum variation sampling was used. Following the principle of data saturation for sample size calculation, recruitment was ceased when data saturation was achieved, that is, when no new information was obtained through interviewing additional participants.^27^

### 3.5 Data collection and analysis

Eligible participants were required to sign a written informed consent form after agreeing to participate. Individual semi‐structured interviews were carried out via telephone by the same researcher in Chinese, and the audio was recorded. Guided by social cognitive theory, three types of interview guides specifically targeted at three groups of participants were used (Supporting Information: Appendix SI), asking questions about rural females' cervical cancer screening self‐efficacy and screening behaviours and the perspectives of service providers.

After each interview, the audio recording was transcribed by a trained research assistant (a registered nurse) into Chinese verbatim for data analysis. Two researchers (M. Z. and T. W.) independently analysed the data through the framework method.^21^ Six main stages via the framework method were adopted: familiarisation, coding, generating a working analytical framework, applying the analytical framework, charting, and interpretation.^21^

Translation (Chinese to English) and back‐translation (English to Chinese) of the study results, including themes, subthemes, and main quotes, were completed by two independent research assistants with master's degrees. Any uncertainty or disagreement during the translation process was discussed to reach a consensus.

### 3.6 Rigor

To assess the findings, four triangulation processes were adopted: method, investigator, theory, and data source.^28^ Credibility, dependability, confirmability, and transferability were considered to ensure trustworthiness.^29^ For credibility, maximum variation sampling was used to select participants with varied experiences to ensure diverse perspectives were included.^30^ To ensure study dependability, semi‐structured interview guides were used, and participants were required to attend the interview in private places alone. Additionally, research notes were used to record the entire study process. To increase confirmability, memos and diaries were used to record all steps carried out during the analysis.^31^ To ensure study transferability, the detailed descriptions of the data were not limited to participants' behaviours and experiences but also included the contexts in which they were reported.^31^

### 3.7 Ethical considerations

The study received ethics approval from the Survey and Behavioural Research Ethics Committee of The Chinese University of Hong Kong (No. SBRE‐20‐748). Informed consent, anonymity, and voluntary participation and withdrawal were ensured.

## 4 RESULTS

### 4.1 Participants

This study was conducted from June to July 2021. Initially, 19 interviews (with interviewees including 15 rural females, two healthcare professionals, and two village staff) were completed. At the point of completing the 19th interview, no new information was obtained. To ensure data saturation, we conducted a further three interviews (one rural female, one healthcare professional, and one village staff) and confirmed that no new information emerged from the interviews. In total, 22 participants (16 rural females, three healthcare professionals, and three village staff) were included in this study.

All rural females included in this study were married and had lived in a rural area for at least 15 years. Most participants had an educational level of senior middle school or lower. All had medical insurance. Thirteen rural females were aged over 35 years and were eligible for the national screening programme, and three were aged between 25 and 34 years and were thus not covered by the programme. Half of the participants had a history of cervical cancer screening (Table 1). Most of the healthcare professionals and village staff included in this study had been involved in cervical cancer screening‐related services for at least three years (Table 2). The duration of the interviews ranged from 29 to 63 min.

**Table 1:** Sociodemographic information of rural females received interviews.

Variable	*N*	%
Age		
25–34	3	18.75
35–44	6	37.50
45–54	5	31.25
55–64	2	12.50
Marital status		
Single/divorced/widowed	0	0
Married	16	100
Resident duration in rural areas		
≤15 years	0	0
>15 years	16	100
Education background		
Primary school or lower	2	12.50
Junior middle school	6	37.50
Senior middle school	2	12.50
Vocational colleges	3	18.75
Junior college	3	18.75
Occupation		
Housewife	5	31.25
Staff of enterprises	4	25
Migrant worker	2	12.50
Self‐employed	5	31.25
Household monthly average income		
1000–3000 CNY per person	6	37.50
3001–5000 CNY per person	10	62.50
Have medical insurance		
Yes	16	100
No	0	0
Cervical cancer screening history		
At least once during the past 3 years	6	37.50
Many times, but not received during the past 3 years	0	0
Only once before but not received during the past 3 years	2	12.50
Never received	8	50

**Table 2:** Working information of healthcare professionals and village staff received interviews.

Variable	*N*	%
*Healthcare professionals*		
Occupation		
Doctor	3	100
Nurse	0	0
Age		
25–34	0	0
35–44	3	100
Duration of employment		
5–10 years	2	66.67
11–15 years	1	33.33
Duration of involved in cervical cancer screening service		
5–10 years	3	100
>10 years	0	0
*Village staff*		
Age		
25–34	2	66.67
35–44		33.33
Education background		
Vocational colleges	1	33.33
Junior college	2	66.67
Times for participating in national screening programme		
1–3 times	1	33.33
4–6 times	2	66.67

### 4.2 Sources of cervical cancer screening self‐efficacy among rural females

The sources of cervical cancer screening self‐efficacy identified among rural females in this study were grouped according to the four major determinants (performance accomplishment, vicarious experience, verbal persuasion, and emotional arousal) defined by social cognitive theory.^22^

#### 4.2.1 Category 1: Personal cervical cancer screening experience

Rural females' personal cervical cancer screening experience, mainly including two aspects: personal screening history and personal perception of screening, showed varied influence on their screening‐related self‐efficacy.

##### Subcategory 1: Screening history contributing to the understanding of cervical cancer screening

We found that compared with those without, some rural females with a screening history showed a deep understanding of cervical cancer screening and held a positive attitude toward it.

Most rural females reported that after undergoing their first cervical cancer screening, they more clearly understood what the screening involved and its purpose. After experiencing the screening process, they considered it easy and essential. These rural females with a screening history showed a positive attitude towards cervical cancer screening and intended to continue undergoing screening in the future.

**Table:** 

**Interviewee**	**Quotation**
*Rural female A (58 years old, married, had screening history)*	*Last year I underwent a cervical cancer screening. The process included registering, making an appointment, and completing the screening. It was simple and convenient… It helps me to know whether I will get cancer or not. I will continue to undergo the screening*.
*Rural female D (44 years old, married, had screening history)*	*I have received cervical cancer screening several times… it was good for my health. I will keep receiving regular screening in the future*.

Rural females without screening history demonstrated a lack of awareness about cervical cancer screening. Abnormal menstruation and pain were the two most unusual symptoms mentioned by them. They believed that if they did not have apparent signs of these two symptoms, they were gynaecologically healthy, and thus, they believed that they did not need to undergo cervical cancer screening. Healthcare professionals similarly reported that rural females relied on menstruation‐related symptoms to estimate their gynaecological health.

**Table:** 

Interviewee	Quotation
*Rural woman L (42 years old, married, no screening history)*	*I have no irregular menstruation, and I have not experienced any discomfort in my body, so I guess I don't need to receive these unnecessary gynaecological examinations, like cervical cancer screening*.
*Healthcare professional B*	*Women in their fifties are in their perimenopause or menopause… They no longer have regular menstruation, and due to this their attention and awareness of their gynaecological health have naturally decreased. They simply believe that as menstruation starts to disappear, the possibility of having gynaecological illness also substantially decreases*.

##### Subcategory 2: Misunderstanding impeding receiving cervical cancer screening

One noteworthy finding in our study was that, although some rural women had already undergone a screening, they still demonstrated a misunderstanding of cervical cancer screening. Some believed that the screening was a one‐time examination. As their screening results were normal, they judged themselves as free from cervical cancer and believed there was no need to undergo screenings in the future.

**Table:** 

Interviewee	Quotation
*Rural female C (49 years old, married, had screening history)*	*If the results were abnormal, I would insist that the screening was repeated per the doctor's suggestion. However, as the screening results were normal, I feel there is no need to continue undergoing screenings*.
*Village staff C*	*Some rural females said that receiving the screening once was enough. As they received a ‘safe’ result at the time, they believed they did not need screening again*.
*Healthcare professional B*	*Some rural females completed the screening, and the results were normal. Many of them were confused about why the screening needs to be repeated in the future. They believed once was enough, and that it was not necessary to continue*.

Meanwhile, for those rural women lacking screening history, we found their misunderstanding of cervical cancer screening mainly related to stigma. They believed having a gynaecological disease was shameful and equalled having a history of indiscreet sexual behaviour. This misunderstanding made them have preconceptions about receiving cervical cancer screening, and this kind of biased cognition led rural women to stand opposite to cervical cancer screening.

**Table:** 

**Interviewee**	**Quotation**
*Rural female L (42 years old, married, no screening history)*	*Gynaecological examinations are considered unusual, and it is better not to let others know because people may assume you have some shameful sex‐related disease*.
*Healthcare professional A*	*Many rural women thought only ‘unclean’ sexual behaviour would lead to gynaecological diseases. To prove to others that they did not engage in degenerate sexual behaviours and that they were completely healthy, they refused to undergo screening*.
*Village staff B*	*For some women… in their understanding, having a gynaecological examination was disgraceful. They wrongly believed that cervical cancer screening aimed to detect sex‐related diseases and that having a sex‐related disease meant having indiscreet sexual behaviours*.

These findings suggest that relevant personal experiences influenced rural women's cervical cancer screening‐related self‐efficacy via two processes: the accomplishment of cervical cancer screening and the internalisation of screening. The accomplishment of screening refers to rural women's completion of the screening, and the internalisation of screening refers to rural women's understanding of cervical cancer and screening. The accomplishment of screening was not strongly associated with enhanced cervical cancer screening self‐efficacy, as many rural women with a screening history still believed that repeated screening was unnecessary. Meanwhile, rural women's misunderstandings of cervical cancer and screening decreased their related self‐efficacy.

#### 4.2.2 Category 2: Hearing about other women's cervical cancer screening experiences

In this study, rural females mainly discussed their female relatives', friends', and neighbours' cancer screening‐related experiences and how these influenced their own attitudes towards cervical cancer screening. They commonly reported that ‘my family/friends/neighbours underwent screening first, so I considered undergoing cervical cancer screening too’.

**Table:** 

**Interviewee**	**Quotation**
*Rural female E (40 years old, married, had screening history)*	*My elder sister underwent cervical cancer screening. She told me she had experienced some discomfort and symptoms and the doctor suggested she undergo the screening. I used to experience similar symptoms. So, I realised that I should undergo a screening as well. Therefore, I went to the hospital and got a screening*.
*Rural female B (51 years old, married, had screening history)*	*I knew some of my neighbours and friends had undergone cervical cancer screening through the national screening programme. Because they completed the screening, I felt it was also necessary for me to undergo a screening*.

Additionally, we found that rural females' acceptance of cervical cancer screening was increased if they underwent the screening with a companion. Their companions' decision to undergo a screening too directly influenced the rural females' choices. They also tended to undergo the screening to stay aligned with their companions.

**Table:** 

Interviewee	Quotation
*Rural female F (38 years old, married, had screening history)*	*I underwent cervical cancer screening with my colleagues. All female colleagues in the company chose to receive cervical cancer screening, so I chose to complete it with them together*.
*Rural female D (44 years old, married, had screening history)*	*My neighbours and I participated in a health talk about cervical cancer screening together. I asked her, ‘do you plan to receive cervical cancer screening?’. She said, ‘of course, I will’. So, we decided to complete the cervical cancer screening together*.

This suggests that rural females' screening self‐efficacy could be enhanced by observing, comparing, and benefiting from the screening behaviours of close acquaintances. Rural females may not understand whether they should undergo cervical cancer screening. Observing other individuals' screening experience, and then comparing themselves' similar contexts with these people, rural females could benefit from this process. Their awareness of their own eligibility to undergo the screening could be increased.

#### 4.2.3 Category 3: Professional health education and consultation

Public health education was a significant factor influencing rural females to seek cervical cancer screening‐related information. In this study, rural females, healthcare professionals, and village staff frequently mentioned the impact of public health education. Additionally, interpersonal information‐sharing and personal hospital visits were reported by rural females as a significant path to being referred for screening. Professional health education and consultation prompted rural females to undergo cervical cancer screening.^32^

##### Subcategory 1: Public health education provided by healthcare professionals

In mainland China, with the implementation of the national screening programme, relevant public health education activities were frequently held in rural villages.^14^ In this study, many rural females with a history of screening mentioned that they knew about cervical cancer screening due to participating in the public health education services provided through the national screening programme. Healthcare professionals and village staff reported that such public health promotion could increase rural females' involvement in the programme and could be regarded as a facilitator of their cervical cancer screening self‐efficacy.

**Table:** 

**Interviewee**	**Quotation**
*Rural female D (44 years old, married, had screening history)*	*My neighbours and I attended a health talk held in our community clinic. They told us screening was good for our health. So, I underwent a screening after that*.
*Healthcare professional C*	*From my perspective, health education activities, such as health talks provided by us, were a significant promotor of rural females' participation in screening*.
*Village staff C*	*The feedback on these educational activities from rural females was generally good. Rural females were willing to join in the activities. They are interested in receiving health‐related knowledge*.

##### Subcategory 2: Information conveyed by acquaintances

Information‐sharing by others was identified to be a vital channel through which rural females obtain public health education. For many rural females with a history of screening, after they accessed screening‐related services, they shared their experiences and related information with their female relatives, friends, and neighbours. Several rural females with screening history reported that they would like to share such related information with their close acquaintances.

**Table:** 

**Interviewee**	**Quotation**
*Rural female D (44 years old, married, had screening history)*	*After my neighbours and I underwent screening, we returned home together and told our other neighbours about the national screening programme. I introduced the programme to them and encouraged them to undergo screening. Some of them went to the clinic and completed the screening the next day*.
*Rural female G (29 years old, married, had screening history)*	*After I was told about cervical cancer screening by the doctor, I thought my mother should also receive the screening, as she was 57 years old but had never undergone a screening before. So, I told her about why we need screening just like the doctor told me. I also suggested that she receive cervical cancer screening*.
*Healthcare professional B*	*Some females told me that their neighbours recently participated in the national screening programme, and that these neighbours persuaded them to come here and receive screening too. That is why they knew about the national screening programme and finally underwent a screening*.

We also found that if some rural females, and even some males, lacking the screening history had an opportunity to access screening‐related information, some of them chose to further share this information with their female relatives, friends, and neighbours.

**Table:** 

**Interviewee**	**Quotation**
*Rural female K (32 years old, married, without screening history)*	*When I saw a poster for the national screening programme, I noticed the age limit and knew I was not eligible; however, my mum and aunts were eligible. I told them about the programme and persuaded them to use this opportunity to receive cervical cancer screening*.
*Village staff C*	*When we hold health talks, some males would walk by and stop and ask for leaflets to bring back home for their wives*.

Moreover, both healthcare professionals and village staff reported that, from their perspectives, information‐sharing among rural females was an extremely significant path by which screening‐related information was shared.

**Table:** 

**Interviewee**	**Quotation**
*Healthcare professional A*	*The power of marketing among the rural females themselves is huge, and we cannot ignore it… they (rural females) can spread information about the screening to their relatives and acquaintances*.
*Village staff B*	*Information‐sharing among the rural females themselves is important as this helps to ensure that the information reaches even more people*.

##### Subcategory 3: Private health consultations with healthcare professionals

Private health consultations with healthcare professionals were found to be another major way in which screening‐related information and suggestions reached rural females. When rural females visited healthcare facilities to seek medical help, some would receive advice from healthcare professionals to undergo cervical cancer screening. In this study, some rural females mentioned that their personal clinical visit history was the reason why they underwent screening.

**Table:** 

**Interviewee**	**Quotation**
*Rural female C (49 years old, married, had screening history)*	*I went to see a doctor because I had some discomfort. The doctor told me to undergo a cervical cancer screening and explained the reasons. Then, I followed her advice and received the screening*
*Rural female G (29 years old, married, had screening history)*	*The doctor suggested that, at my age, I should consider starting the cervical cancer screening. I contemplated this for a while and finally decided I should get this examination*.

#### 4.2.4 Category 4: Emotional status

**Figure 1:**
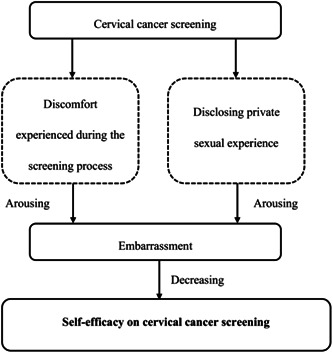
Emotional status.

Individuals' emotional states impact their self‐efficacy, with different negative or positive emotions affecting it in variable ways. We found that rural females' emotional arousal was mainly related to the embarrassment they experienced in relation to cervical cancer screening. We identified two forms of embarrassment experienced by rural females in response to cervical cancer screening that negatively influenced their screening self‐efficacy (Figure 1).

##### Subcategory 1: Embarrassment about discomfort experienced during the screening process

The first form of embarrassment identified among rural females in response to screening was related to the physical discomfort experienced when healthcare professionals performed the screening, and their shame of exposing their genitalia. Some rural females complained about discomfort during the screening process, with ‘pain’ and ‘feeling awkward’ being commonly mentioned.

**Table:** 

**Interviewee**	**Quotation**
*Rural female C (49 years old, married, had screening history)*	*I experienced a lot of pain when the screening started. And embarrassed. Very, very embarrassed. It was only the doctor and I in the room, and I was nearly nude. That was so bad… and to be honest, I do not want to experience this screening again*.
*Rural female F (38 years old, married, had screening history)*	*I felt very uncomfortable. I had to take off my pants and show the most private part of my body to a person I had never met before. That was very awkward and even made me feel a little scared*.

##### Subcategory 2: Embarrassment about disclosing sexual history

The second form of embarrassment identified was related to having to disclose sexual history when receiving cervical cancer screening. Females aged over 25 years with a sexual history need to undergo cervical cancer screening.^33^ Healthcare professionals mentioned that unmarried rural females, in particular, experienced this kind of embarrassment. Receiving the screening meant admitting that they had had premarital sex. To hide this, unmarried rural females avoided undergoing cervical cancer screening.

**Table:** 

**Interviewee**	**Quotation**
*Healthcare professional A*	*Some females refused screening because they were unmarried. I think for young unmarried women, it is really embarrassing to admit having a sexual experience*.
*Healthcare professional C*	*For some young unmarried females, they were unwilling to undergo screening despite being eligible. Perhaps they did not want us to know that they had a sexual history. Perhaps they felt uncomfortable discussing this with us*.

One married rural female mentioned that before she was married, although she had already had sexual experiences, she just ‘pretended not to have sexual experience and thus not be eligible for some gynaecological examinations’, because she felt embarrassed to let others know she had sexual experience.

**Table:** 

Interviewee	Quotation
*Rural female O (34 years old, married, had screening history)*	*It was normal for me to refuse these kinds of examinations at that time. I felt uncomfortable saying yes when they asked me whether I had sexual experience. I was unmarried, and I did not want unfamiliar people to know the details of my sex life. I found that so odd and embarrassing*.

However, we found that the influence of negative emotions exerted on rural females' screening‐related self‐efficacy was not very strong. Rural females mentioned that although they felt uncomfortable with the screening process, if they were experiencing health issues and healthcare professionals suggested they undergo screening, and they would follow their advice.

**Table:** 

**Interviewee**	**Quotation**
*Rural female F (38 years old, married, had screening history)*	*I understand the need for screening. If the doctor asks me to undergo the screening, I will do so. I can overcome feelings of discomfort because my health is the most important thing*.

## 5 DISCUSSION

Based on the social cognitive theory, this qualitative study used framework method to explore sources of cervical cancer screening self‐efficacy among rural Chinese females.^22^ Generally, the main sources of rural females' cervical cancer screening self‐efficacy identified could be divided into four categories: personal experience of cervical cancer screening, experiences of close acquaintances, professional health education and consultation, and emotional state. These four categories align with the four main sources of self‐efficacy proposed by social cognitive theory.

We found that rural females' personal cervical cancer screening experiences contributed to their self‐efficacy through two processes: the accomplishment of screening itself and the internalisation of the screening experience. The former refers to rural females' completing the screening, while the latter refers to their understanding and cognition of cervical cancer screening. For rural females without screening history, we found they believed a lack of relevant symptoms (e.g., pain or abnormal menstruation) meant they were healthy and did not need any gynaecological examinations. A study that surveyed rural Nepalese females' knowledge of and attitudes towards cervical cancer screening reported that they lacked screening‐related knowledge, and that most believed no relevant symptoms meant no need for cervical cancer screening.^34^ A lack of screening behaviour and cognition negatively impacted the screening self‐efficacy of these rural females. However, we also found that rural females with screening history still showed insufficient self‐efficacy on repeat screening. This indicates that undergoing a cervical cancer screening alone does not strongly enhance rural females' screening self‐efficacy. Benefiting from the national screening programme, many rural Chinese females can access free cervical cancer screening. In this study, rural females reported that this free service contributed to them being willing to undergo cervical cancer screening. However, this mainly encouraged them to gain experience of ‘completing the screening behaviour’, while the experience of ‘understanding and internalising the screening’ was not sufficiently obtained. A study of teachers' teaching self‐efficacy proposed two forms of mastery experiences (i.e., experience accomplishments): enactive mastery and cognitive mastery.^35^ Enactive mastery relates to individuals' acts and the successful completion of specific tasks. In contrast, cognitive mastery refers to the successful understanding of something rather than the act of doing something, which relates to individuals' cognitive processing of the task.^26^ Another study reported that cognitive mastery experience had a more significant influence on individuals' self‐efficacy than enactive mastery experience, and proposed that enactive mastery alone was not enough to significantly impact individuals' self‐efficacy.^25^ Our findings were consistent with their results. Rural females' experiences of ‘completing the screening behaviour’ could be regarded as enactive mastery, while their experiences of ‘internalising the screening’ represent cognitive mastery. In this study, some rural females with screening history gained enactive mastery, but still lacked cognitive mastery. In their cognition, they considered cervical cancer screening as a ‘one‐time’ examination, so they believed regular screening was not necessary. Due to the lack of cognitive mastery, undergoing the screening (i.e., the enactive mastery) was not sufficient to significantly enhance rural females' cervical cancer screening self‐efficacy. This could explain why, in this study, we found that several rural females with the screening history still demonstrated a deficient awareness of the need for regular screening. Compared with enactive mastery, cognitive mastery of cervical cancer screening was more influential in improving rural females' screening self‐efficacy. One study revealed the relationship between women's cervical cancer screening‐related knowledge and self‐efficacy. This research reported that increasing knowledge can influence the increment of women's cervical cancer screening self‐efficacy.^36^ Thus, rural females' lack of cognitive mastery was an important factor. Therefore, considering improving rural women's cognitive abilities to enhance their self‐efficacy, health education could be considered implemented. Helping rural females to gain a sufficient understanding of the need for regular cervical cancer screening, and thus gain cognitive mastery, should be emphasised when conducting cancer screening‐related education in the future.

As a vicarious experience, hearing about the cervical cancer screening experiences of other close acquaintances also significantly improved rural females' screening self‐efficacy. Relatives', friends', and neighbours' cervical cancer screening experiences could influence rural females' screening‐related self‐efficacy. For rural females, these acquainted people are in the ‘high‐trust’ level of their interpersonal network. Because of this trust, rural females were willing to accept their suggestions and mimic their behaviours. A study that examined vaccine hesitancy found that people trusted comments from their families and friends more than those from unfamiliar individuals.^37^ This result demonstrated that individuals' health‐related behaviours can be influenced by those in a close relationship with them.^38^, ^39^ Relatives', friends', and neighbours' previous screening experiences can thus be regarded as an encouragement for rural females. This encouragement might increase rural females' own interest, awareness, and willingness to receive cervical cancer screening and compel them to undergo screening by imitating familiar people's behaviour. Therefore, to enhance rural females' screening‐related self‐efficacy, the significant influence of interpersonal relationships should be exploited. Given this, we propose that community‐based interventions should be considered in the future. The engagement and performance of other people within the community could influence other rural females. The WHO has also emphasised that community outreach and mobilisation are fundamental when conducting cervical cancer‐related health education.^11^ Studies that implemented community‐based interventions for promoting cervical cancer screening among rural females have similarly reported that such interventions conducted in a community environment were feasible and effective.^40^, ^41^, ^42^

Public health education and promotion provided by healthcare professionals, the information conveyed by close acquaintances, and private health consultations from healthcare professionals served as verbal persuasion and helped to enhance rural females' screening‐related self‐efficacy. Social cognitive theory proposes that verbal persuasion is a commonly used method of influencing individuals' self‐efficacy. However, the impact of verbal persuasion on self‐efficacy is likely weaker than that of experience accomplishment.^22^ However, in this study, we found that various forms of verbal persuasion were commonly mentioned by rural females and screening service providers, and they regarded them as ‘vital channels for receiving screening‐related information’. They agreed that these kinds of verbal persuasion had a vitally persuasive influence on rural females' screening‐related self‐efficacy. In parallel with the national screening programme, related public health education initiatives have been implemented, which have increased the social awareness of cervical cancer screening.^14^ In line with this, we found that the influence of verbal persuasion on rural females' screening‐related self‐efficacy was profound. Thus, to enhance rural females' screening self‐efficacy, the effect of verbal persuasion should be considered when designing interventions in the future.

Several rural females reported being embarrassed when receiving cervical cancer screening. We found that rural females mainly experienced two forms of embarrassment, consistent with the findings of a previous study.^43^ Such screening‐associated embarrassment was mainly related to pain, awkwardness, or even fear. Both fear and embarrassment were common emotional responses to cervical cancer screening.^44^ A study similarly reported that some rural females complained of discomfort or pain during cervical cancer screening.^45^ Another study reported that some females believed that relaxing their bodies could help make the screening easier and less painful.^46^ Therefore, providing a detailed introduction to the screening process and encouraging the use of relaxation techniques during screening could ease these feelings of discomfort.^47^ The second form of embarrassment commonly reported was related to having to discuss prior sexual experiences with others. We found that unmarried rural females tended to be ashamed to discuss their sexual experiences. They were resistant to receiving gynaecological examinations targeted at females who had sexual experiences. This is likely related to the taboo of premarital sex in rural Chinese social norms and culture. Influenced by traditional culture, rural Chinese people are typically more conservative about premarital sex than urban Chinese people,^48^ believing that premarital sex is dishonourable. Therefore, without building a marriage relationship, some unmarried rural females felt embarrassed about discussing their sexual experiences, thus influencing their cervical cancer screening‐related self‐efficacy. Our findings agree with those of some previous studies conducted in Asian cultural contexts. For example, one study in Korea reported that due to sexuality being considered an inappropriate topic to discuss, unmarried females did not pursue cervical cancer screening.^49^ One study in Malaysia reported that unmarried females were less likely to undergo cervical cancer screenings because premarital sex is not acceptable in the local culture.^50^ One study in Bahrain found that only a few females showed the intention of undergoing cervical cancer screening if they were unmarried.^51^ Meanwhile, one study conducted among married females in Indonesia demonstrated that receiving family/husband support was a significant factor in their cervical cancer screening‐related attitude.^36^ Negative emotions tended to reduce rural females' cervical cancer screening self‐efficacy.^52^ However, compared with other sources, the influence of emotional status on rural females' screening‐related self‐efficacy was not as significant in our study. Although they felt embarrassed, several rural females highlighted that they could overcome these negative emotions and undergo the screening if their health condition required it. Future studies should explore how to help rural females overcome such negative emotions relating to cervical cancer screening.^53^ Interventions need to focus on eliminating the sociocultural stigma of premarital sex and its relationship with cervical cancer screening.

## 6 STRENGTHS AND LIMITATIONS

This study has two main strengths. First, we recruited both cervical cancer screening service users and providers as participants. This helped us to explore the relevant information comprehensively and thoroughly from multiple perspectives. Second, we used the social cognitive theory as a framework to build the interview guides and structure and analyse the interview data. This theory is comprehensive and commonly used to understand changes in individuals' self‐efficacy.

However, this study also has several limitations. First, this study was based on the rural contexts of mainland China. Considering the sociocultural differences extant among countries, the transferability of the findings to other countries might be limited. To improve transferability, in the description of the findings, we provided rich information relating to the rural Chinese background and the participants' characteristics. Second, during interviews, some questions were related to participants' previous experiences, for which they needed to recall past situations. As a result, bias may have been introduced due to the unreliability of memory. Third, this study aimed to recruit rural females aged 25–64 years, and all recruited participants were married. Early marriage is a social issue in rural China. In some studies of married rural females, the youngest age at marriage was ∼20 years.^54^, ^55^ Notably, cervical cancer screening is primarily targeted at females with sexual experiences, and premarital sex is a sensitive topic in rural China.^48^ Two unmarried rural females were contacted during participant recruitment, but both rejected our invitation to participate in this study. Therefore, in this study, we asked married females to recall their situation before marriage and asked service providers to discuss unmarried rural females' situations.

## 7 IMPLICATIONS

Interventions targeted at enhancing rural females' cervical cancer screening‐related self‐efficacy could be developed based on the major sources of self‐efficacy identified in this study. Encouraging rural females to seek an understanding of cervical cancer screening and enhance their cognitive mastery is necessary. This could help them to improve their screening self‐efficacy. Vicarious experience of close acquaintances of rural females were significantly influential. Therefore, community‐based interventions involving close interpersonal relationships should be considered. Public health education programmes were a common channel through which rural females obtained verbal persuasion, and thus, interventions should be implemented in parallel to, and make use of, the extant public health education programmes.

## 8 CONCLUSION

In this study, we identified the sources of rural females' cervical cancer screening self‐efficacy. These were consistent with the four major sources of self‐efficacy proposed by the social cognitive theory, including personal cervical cancer screening experience, cervical cancer screening experiences of close acquaintances, professional health education and consultation, and emotional status. The personal screening experiences included both enactive and cognitive experiences. We found that enactive experiences alone were not sufficient to significantly enhance rural females' cervical cancer screening self‐efficacy, whereas cognitive experiences played a critical role in enhancing such self‐efficacy. Emotional status mainly involved rural females' embarrassment due to the cervical cancer screening process and negatively influenced their screening self‐efficacy. However, rural females could overcome these negative emotions if their health condition required it. To enhance rural females' cervical cancer screening self‐efficacy and increase screening rates, interventions should be developed and implemented according to these sources of information and our recommendations.

## AUTHOR CONTRIBUTIONS

All authors contribute to the conceptualisation, methodology, validation, formal analysis, and manuscript writing and review of the study. Mengyue Zhang also conducted the study investigation. Janet W. H. Sit and Carmen W. H. Chan were responsible for the study supervision.

## CONFLICT OF INTEREST STATEMENT

The authors declare no conflict of interest.

## Supporting information

Supporting information.

## Data Availability

The data that support the findings of this study are available from the corresponding author upon reasonable request.
