# Supplementary material for: Exploring the sources of cervical cancer screening self‐efficacy among rural females: A qualitative study
Source: Health Expect. 2023 Aug 7;26(6):2361–73. doi: 10.1111/hex.13840 (PMC10632632; doi:10.1111/hex.13840)
Supplement: Supplementary file 1 — Supporting information. [file HEX-26--s001.docx]

# Appendix

## Interview guide for rural females (English version)

**Part I: Basic information**

1. **Age: __________** years old
2. **Marital status:**

| A. Unmarried | B. Married |
| --- | --- |
| C. Divorced | D. Widowed |

1. **Duration of settled in rural areas:**

| A. Less than 5 years | B. 5-10 years |
| --- | --- |
| C. 11-15 years | D. More than 15 years |

1. **Educational level:**

| A. Primary school or lower | B. Junior middle school |
| --- | --- |
| C. Senior middle school | D. Vocational secondary school |
| E. Diploma | F. Bachelor or higher |

1. **Occupation: __________**
2. **Family income per month:**

| A. Less than 1000 CNY per person | B. 1000-3000 CNY per person |
| --- | --- |
| C. 3001-5000 CNY per person | D. More than 5000 CNY per person |

1. **Medical insurance:**

| A. National Health Insurance | B. Commercial Health Insurance |
| --- | --- |
| C. Both national and commercial | D. National employee Insurance |
| E. No medical insurance |  |

1. **Cervical cancer screening history:**

| A. Have received at least once during the past 3 years |
| --- |
| B. Had received many times before, but has not received during the past 3 years |
| C. Had received only once before, but has not received during the past 3 years |
| D. Have never received |

**Part II: Self-efficacy and cervical cancer screening behaviour**

1. **Could you talk about what do you know about cervical cancer and its screening?**

(Prompt: Can you talk about any benefit/harm of cervical cancer? Can you talk about any benefit/harm of cervical cancer screening? How do you get this information?)

1. **Could you talk about your perceptions and feelings about receiving cervical cancer screening?**

(Prompt: To what extent you think it is important for women to get cervical screening. Why do you think so?)

1. **Could you talk about the situation of receiving cervical cancer screening among other females around you (like your families/friends/acquaintance)?**

(Prompt: Could you know why did they receive screening or not? Would their choice of receiving screening or not influence you?)

1. **If you have received cervical cancer screening at least once before:**
2. Could you talk about why you decided to receive screening?
3. Could you talk about some barriers you thought would prevent you to receive screening?

(Prompt: How did you overcome these barriers? What was the biggest barrier?)

1. Could you talk about some facilitators you thought would encourage you to receive screening?

(Prompt: How did you utilize these facilitators? What was the biggest facilitator?)

1. Could you talk about your last cervical cancer screening?

(Prompt: When did you receive it? Where did you receive it? What kind of screening method did you get? How about the result? How do you feel during the whole process?)

(5) Could you talk about your plan about receive cervical cancer screening in the future?

1. **If you never received cervical cancer screening before:**
2. Could you talk about why you did not receive screening?
3. Could you talk about some barriers you thought would prevent you to receive screening?

(Prompt: Why did you not overcome these barriers? What was the biggest barrier?)

1. Could you talk about some facilitators you thought would encourage you to receive screening?

(Prompt: Why did you not utilize these facilitators? What was the biggest facilitator?)

1. Could you talk about have you planned to receive cervical cancer screening before?

(Prompt: Why did you not receive it as planned?)

1. Could you talk about your plan about receive cervical cancer screening in the future?

## Interview guide for professional healthcare professionals (English version)

**Part I: Basic information**

1. **Occupation: __________**
2. **Age: __________** years old
3. **Duration of employment:**

| A. Less than 5 years | B. 5-10 years |
| --- | --- |
| C. 11-15 years | D. More than 15 years |

1. **Duration of involved in cervical cancer screening service：**

| A. Less than 5 years | B. 5-10 years |
| --- | --- |
| C. 11-15 years | D. More than 15 years |

**Part II: Cervical cancer screening of rural females**

1. **Could you introduce the local resources/services of cervical cancer screening?**

(Prompt: How about the situation of the National Cervical Cancer Screening Program (NCCSPRA)? Besides the NCCSPRA, is there any other activities related to cervical cancer screening locally? How about their effects?)

1. **Could you talk about the feedback of the local resources/services of cervical cancer screening from rural females who need to receive cervical cancer screening (including women covered by the NCCSPRA or not, which means females aged 25-64 years)?**

(Prompt: How their attitude towards receiving cervical cancer screening How about their feelings and experience about the local resources/services of cervical cancer screening?)

1. **From your perspective, for rural females need to receive cervical cancer screening (including women covered by the NCCSPRA or not, which means females aged 25-64 years), could you introduce to what extent they understand cervical cancer and its screening?**
2. **Could you talk about the situation of cervical cancer screening among rural women who are included in the NCCSPRA (women aged 35-64 years)?**

(Prompt: How about the official screening uptake rate?)

1. **Could you talk about the situation of cervical cancer screening among rural women who also have necessity of receiving screening but not included in the NCCSPRA (women aged 25-34 years)?**

(Prompt: How about the official screening uptake rate?)

1. **From your perspective, could you talk about reasons why rural females (including women covered by the NCCSPRA or not, which means women aged 25-64 years) receive cervical cancer screening or not?**
2. **From your perspective, for cervical cancer screening, what do you think are barriers/facilitators to cervical cancer screening of rural females (including women covered by the NCCSPRA or not, which means women aged 25-64 years)?**

(Prompt: What is the biggest barrier/facilitator? Why do you think so?)

## Interview guide for village staff (English version)

**Part I: Basic information**

1. **Occupation: __________**
2. **Age: __________** years old
3. **Educational level:**

| A. Primary school or lower | B. Junior middle school |
| --- | --- |
| C. Senior middle school | D. Vocational secondary school |
| E. Diploma | F. Bachelor or higher |

1. **Times of participating the health education and promotion of the National Cervical Cancer Screening Program (NCCSPRA) in Rural Areas**

| A. 1-3 times | B. 4-6 times |
| --- | --- |
| C. 7-9 times | D. 10 times or more |

**Part II: Cervical cancer screening of rural females**

1. **Could you introduce the health education and promotion of the NCCSPRA?**

(Prompt: How about the main workflow of its health education and promotion? What are the main formats and contents of its health education and promotion activities?)

1. **Could you talk about the feedback of health education and promotion of the NCCSPRA from rural females who are included this project?**

(Prompt: How about the effects of education and promotion activities? How their attitude towards participating the NCCSPRA?)

1. **From your perspective, about these rural females who are included in the NCCSPRA, could you introduce to what extent they understand cervical cancer and its screening?**
2. **Could you talk about the situation of cervical cancer screening among rural women who are included in the NCCSPRA?**

(Prompt: How about the official screening uptake rate?)

1. **From your perspective, why these rural females who are included in the NCCSPRA choose to receive cervical cancer screening or not?**
2. **From your perspective, for cervical cancer screening, what do you think are barriers/facilitators to cervical cancer screening of rural females?**

(Prompt: What is the biggest barrier/facilitator? Why do you think so?)
